# Rescuing SERCA2 pump deficiency: A novel approach to improve bone mechano-responsiveness in type 2 diabetes

**DOI:** 10.1016/j.mbm.2024.100047

**Published:** 2024-02-24

**Authors:** Zhifeng Yu, X. Edward Guo

**Affiliations:** a Shanghai Key Laboratory of Orthopaedic Implants, Department of Orthopaedic Surgery, Shanghai Ninth People's Hospital, Shanghai Jiao Tong University School of Medicine, Shanghai, China; b Bone Bioengineering Laboratory, Department of Biomedical Engineering, Columbia University, New York, NY, USA

**Keywords:** SERCA2 pump, Osteocyte calcium dynamics, Calcium oscillatory, Mechanotransduction

## Abstract

A recent study published in *Nature Communications* demonstrated that restoring SERCA2 pump deficiency can enhance bone mechano-responsiveness in type 2 diabetes (T2D) by modulating osteocyte calcium dynamics. The findings revealed that in T2D mice, the ability of the bone to respond to mechanical stress is compromised, primarily due to attenuated calcium oscillatory dynamics within osteocytes rather than in osteoblasts or osteoclasts. The underlying mechanism of this reduction in bone mechano-responsiveness in T2D was identified as a specific decrease in osteocytic SERCA2 expression mediated by PPARα. Additionally, mice overexpressing SERCA2 in osteocytes exhibited reduced deterioration of bone mechano-responsiveness induced by T2D. Collectively, this study provides mechanistic insights into T2D-induced deterioration in bone mechano-responsiveness and identifies a promising therapeutic approach to counteract T2D-associated fragility fractures.

Type 2 diabetes (T2D) is a common chronic disease that affects over 400 million people worldwide,^1^ characterized by insulin resistance and persistent hyperglycemia. T2D leads to a range of complications, including fractures being one of them. The risk of fractures is higher in T2D patients than in healthy individuals, posing a substantial burden on healthcare systems.^2^ However, the current treatment options for T2D-related bone loss are limited, necessitating further research to identify novel therapeutic strategies.

The bone is an active tissue that maintains its integrity through mechanical loading. Mechanical stimulation is crucial for preserving the bone mass and quality. Under appropriate mechanical loading, bones adapt to stimuli by increasing the bone mass and improving the bone quality, thereby maintaining a healthy state. Osteocytes are a primitive cell type that can be traced back to the fossilized skeletal remains of ancient fish and dinosaurs.^3^ These cells reside within the bone matrix and possess unique molecular remodeling capabilities, allowing them to modify their surrounding extracellular environment independently of osteoblasts (bone-forming cells)^4^ and osteoclasts (bone-resorbing cells).^5^ Mechanotransduction within osteocytes is an intricate and sophisticated regulatory mechanism that involves complex interactions between cells and their environment, neighboring cells, and distinct functional mechanosensors within individual cells.^6^ This process ensures optimal communication and responses to mechanical stimuli, thereby maintaining bone homeostasis and integrity.

Normally, bones adjust to mechanical loads, a phenomenon known as bone adaptability. However, this adaptability is impaired in T2D. Studies have shown that T2D patients have lower bone quality and an increased risk of fractures.^7^ Furthermore, the response of the bones to mechanical loads is attenuated in patients with T2D. To better understand the impact of T2D on bone mechanical response, researchers conducted a series of experiments. Recently, an intriguing article entitled “Rescuing SERCA2 pump deficiency improves bone mechano-responsiveness in T2D by shaping osteocyte calcium dynamic” published in Nature Communications^8^ revealed that in T2D models, whether spontaneous or experimentally-induced, the bone's response to mechanical loads was impaired. This impairment is related to attenuated calcium ion dynamics in osteocytes, rather than in osteoblasts. Further investigations revealed that the reduction in SERCA2 expression in osteocytes was responsible for attenuated calcium ion dynamics.

SERCAs, which belong to the P-type ATPase family that also includes H^+^/K^+^ ATPases, Na^+^/K^+^ ATPases, and plasma membrane Ca^2+^ ATPases, serve as pumps that catalyze the transport of Ca^2+^ across the membrane using energy derived from ATP hydrolysis.^9^ Accumulating evidence suggests that the dysregulation of Ca^2+^ dependent on SERCA2 plays a role in the development of various cardiovascular and neurodegenerative diseases.^10^^,^^11^ Notably, Morrell AE et al.^12^ observed that cytoskeletal contractions were mediated by Ca^2+^ transients in osteocytes in response to fluid shear stress. Specifically, in contrast to constant Ca^2+^ signaling, oscillatory fluctuations in cytosolic Ca^2+^ levels are advantageous for enhancing the efficiency and specificity of gene expression, thereby exerting control over numerous subsequent cellular processes.

In T2D, reduced SERCA2 expression leads to attenuated calcium ion dynamics in osteocytes, thereby affecting the bone response to mechanical loads. To validate the role of SERCA2 in T2D bone mechanical response, researchers used a SERCA2 agonist called istaroxime for treatment. Istaroxime, a drug that can increase SERCA2 pump activity, was found to improve osteoporosis symptoms by restoring calcium ion oscillatory dynamics in osteocytes. The experimental results showed that the bone architecture and strength of T2D mice treated with istaroxime significantly improved in the context of mechanical loads. Specifically, istaroxime improves the calcium response of osteocytes to mechanical loads, thereby promoting osteoblast activity and inhibiting osteoclast activity.

In addition to drug treatments, researchers have increased SERCA2 expression in osteocytes using transgenic animal models. This approach is another potential option for the treatment of diabetic osteoporosis. By increasing SERCA2 expression, calcium ion oscillatory dynamics in osteocytes can be restored, thereby improving osteoporosis symptoms.

These results indicate that SERCA2 plays a crucial role in the mechanical response of bones in T2D. The lack of SERCA2 can lead to attenuated calcium ion dynamics, thereby affecting the response of the bone to mechanical loads. By restoring calcium ion oscillatory dynamics and increasing the expression of the SERCA2 pump, the symptoms of osteoporosis can be improved, and the risk of fractures can be reduced (Fig. 1). These findings provide new ideas and approaches for the treatment of diabetic osteoporosis, potentially benefitting a wide range of patients with T2D.

**Fig. 1:**
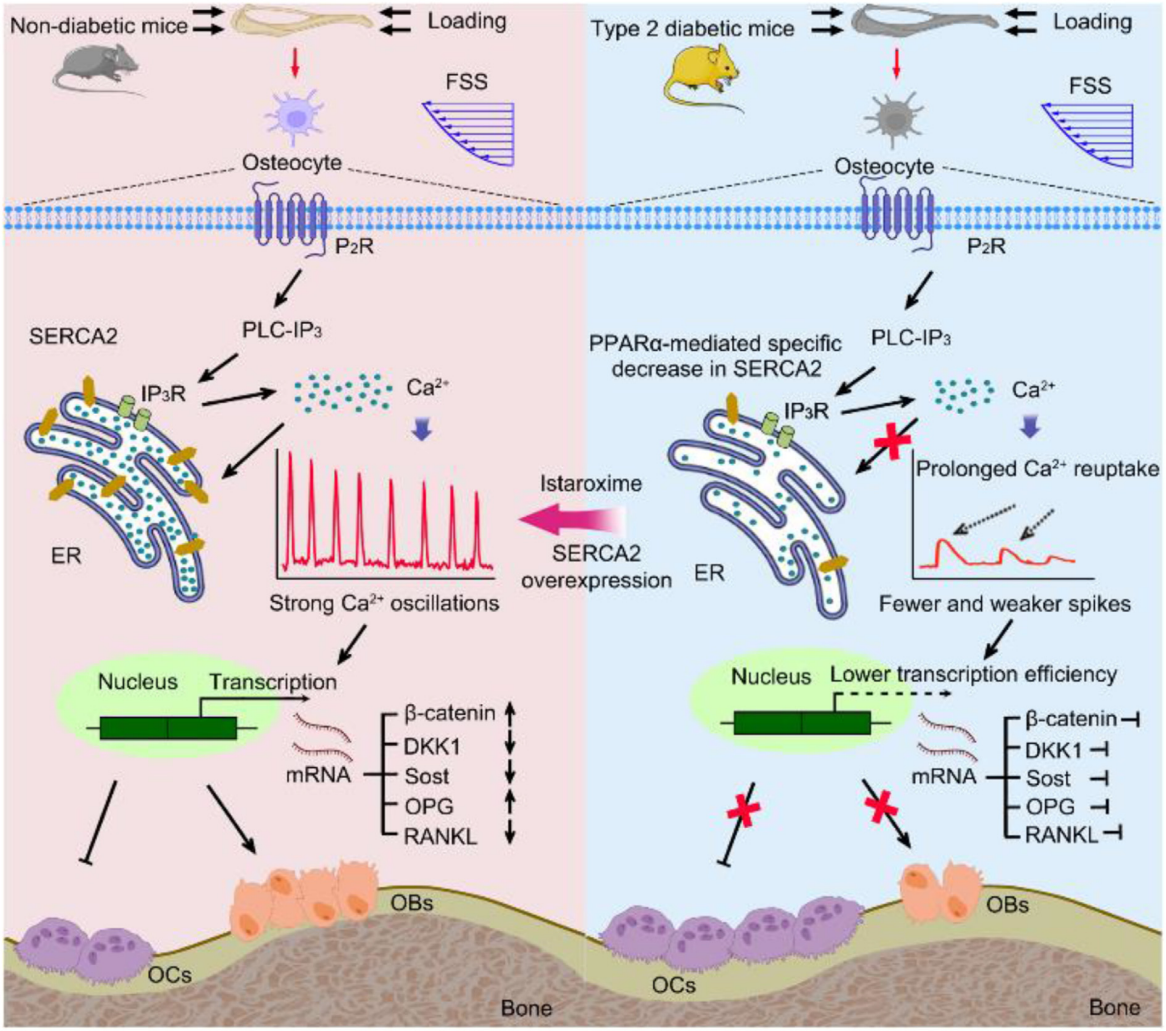
Schematic illustration of how Type 2 Diabetes (T2D) impairs bone mechano-responsiveness through SERCA2 pump deficiency.^8^

This study focused only on the impact of SERCA2 on the mechanical response of bones in patients with T2D. However, the effects of T2D on the bones are multifaceted, including bone density, bone quality, and fracture risk. Therefore, future research should comprehensively explore the impact of T2D on bones and search for additional treatment strategies. Additionally, although this study showed that SERCA2 agonists can improve the mechanical response of bones in T2D, the safety and effectiveness of this treatment approach still needs to be further validated through clinical trials. Further research is required to explore the translation of this potential treatment strategy into practical therapeutic methods.

## Ethical approval

This study does not contain any studies with human or animal subjects performed by any of the authors.

## Declaration of competing interest

The authors declared no potential conflicts of interest with respect to the research, authorship, and/or publication of this article.
